# An evaluation of the adequacy of Indian national and state essential medicines lists (EMLs) for palliative care medical needs—a comparative analysis

**DOI:** 10.3332/ecancer.2025.1837

**Published:** 2025-02-05

**Authors:** Disha Agrawal, Divya Shrinivas, Parth Sharma, Muttacaud Ramakrishnan Rajagopal, Arun Ghoshal, Siddhesh Zadey

**Affiliations:** 1 Association for Socially Applicable Research (ASAR), Pune 411007, Maharashtra, India; 2 Maulana Azad Medical College, New Delhi 110002, India; 3 Trivandrum Institute of Palliative Sciences, Trivandrum, Kerala 695009, India; 4 Department of Palliative Medicine and Supportive Care, Kasturba Medical College and Hospital, Manipal 576104, Karnataka, India; 5 Dr. D. Y. Patil Dental College and Hospital, Dr. D. Y. Patil Vidyapeeth, Pune 411018, Maharashtra, India; 6 Global Emergency Medicine Innovation and Implementation (GEMINI) Research Center, Duke University School of Medicine, Durham, NC 27708, USA; 7 Department of Epidemiology, Columbia University Mailman School of Public Health, New York, NY 10032, USA; † Contributed equally.

**Keywords:** palliative care, palliative medicine, public health, delivery of healthcare, patient care management, morphine, opioid, essential medicines, controlled substances

## Abstract

**Objectives:**

Essential medicines lists (EMLs) guide the public sector procurement and supply of medications to impact access to adequate and appropriate palliative care drugs. This study evaluates the adequacy of India’s national and sub-national EMLs that can directly impact palliative care for 5.4 million patients.

**Methods:**

In this qualitative document review, we compared Indian national, and state EMLs acquired from official government websites with the International Association for Hospice and Palliative Care (IAHPC) EML recommendations. We analysed data on the indication and formulation of drugs under the different categories of formulations present (all, some and no), and drugs absent. Literature review and inputs from palliative care experts provided alternatives of absent medications to assess the adequacy of lists in managing the symptoms listed by IAPHC.

**Results:**

We analysed 3 national and 27 state lists for 33 recommended drugs. The Central Government Health Services list had the maximum availability of all formulations of drugs (16 [48%]) nationally. Among states and union territories, the Delhi EML was the closest to IAHPC with 17 (52%) drugs with all formulations present. Karnataka had the most incomplete EML with only 3 (9%) drugs with all formulations present. No EML had all the recommended formulations of morphine. In one national and seventeen state EMLs, oral morphine was absent.

**Conclusion:**

While Indian EMLs lack drugs for palliative care when compared with the IAHPC EML, symptom management is adequate. There is a need for countries with limited resources to modify the IAPHC list for their settings.

## Introduction

Palliative care deals with reducing serious health-related suffering through the early identification and treatment of physical, psychosocial or spiritual problems related to acute or chronic diseases [1]. Adequate access to and provision of palliative care improves quality of life [2], enables informed treatment decision-making [3] and reduces hospital readmissions and healthcare costs [4]. Although access to palliative care is a right to health globally, only 14% of the 40 million people in need can access it [1].

Although 5.4 million Indians need palliative care annually, merely 1% can access it [5]. In Indian patients with end-stage cancers, the unmet need for palliative care was reported to be 98.3% [6]. Several obstacles disrupt effective palliative care delivery, including poor geographical access, limited awareness, lack of workforce training, restrictive prescription policies for pain medications and limited policy prioritisation, among others [5].

The World Health Organisation (WHO) introduced the concept of essential medicines in 1977 to address priority health needs [7]. These medications are chosen based on their public health importance, efficacy, safety and cost-effectiveness and should be consistently available in sufficient quantities in public health centres. Thus, the implementation of a thoughtfully curated essential medicines list (EML) can enhance the quality of care, management practices and resource allocation, and ensure the availability of medicines by streamlining procurement and distribution processes. While countries can determine their EMLs, the WHO model list serves as a reference for national and institutional lists [8].

India has multiple EMLs. Health is a state subject as per the Indian Constitution. Hence, states draft their EMLs to match the local needs [9]. Two national health insurance schemes – the Employees’ State Insurance Scheme (ESIS) and the Central Government Health Scheme (CGHS) have their EMLs. A third national EML exists for the states without their unique EML to follow. These EMLs guide the procurement of drugs that are dispensed at government-run healthcare institutions at the state and national levels. As the vast majority of Indians reside in rural areas and rely on public (government-run) healthcare facilities, the appropriateness of palliative care service delivery partly depends on the adequacy of the national and state EMLs.

In 2007, responding to a request from the WHO Cancer Control Program, the International Association for Hospice and Palliative Care (IAHPC) collaborated with other organisations to develop a list of essential medicines for the 16 most common palliative care symptoms [10]. The IAHPC is a public charity serving as an international platform to improve access to palliative care and global standards of care [11]. This list, thus, became the model list to serve as a reference for nations globally.

The objective of this study was to evaluate the adequacy of India’s EMLs at the national and state/Union Territory (UT) levels by comparing Indian lists with the list curated by IAHPC. By identifying alternatives to missing drugs in the national and state lists, we also aimed to assess the adequacy of the lists for managing common palliative care symptoms listed by IAHPC.

## Methods

### Data sources

The National List of Essential Medicines (NLEM), last updated in 2022, was designed by the Ministry of Health and Family Welfare, Government of India [12]. The ESIS EML caters to the population employed in factories and other establishments such as hotels, shops and restaurants [13]. The CGHS covers current employees and pensioners of the national (central) government [14]. The state/UT lists were designed for the respective state-level public health facilities.

The IAHPC EMLs were drafted through the consensus of international physicians and pharmacologists. After identifying the most common symptoms in palliative care, a final list of appropriate medications was devised using a modified Delphi process [15]. The IAHPC list included 33 essential drugs, which were looked for in individual national and state EMLs. We accessed the most recent versions of three national and 27 state/UT EMLs from the government websites (Table 1). The links to access the individual EMLs are provided in Supplementary Table 1. The EMLs of 4 states (Arunachal Pradesh, Goa, Sikkim Uttar Pradesh) and 5 UTs (Andaman and Nicobar Islands, Chandigarh, Dadra and Nagar Haveli and Daman and Diu, Ladakh and Lakshadweep) were unavailable.

### Data extraction

We followed the readying material, extracting data, analysing data and distilling findings approach to evaluate national and state (including union territories) EMLs and compared them with the IAHPC EML of essential medicines for palliative care [16]. The drugs’ names and formulations present in the Indian EMLs were compiled in a Microsoft Excel spreadsheet and matched with the IAHPC list to assess their adequacy.

If the EML did not have the drug, we looked for alternative drugs that may be prescribed for the same indication (Table 2). Acceptable alternatives were determined by reviewing the literature and by consulting two experienced palliative care experts (MRR with 31 years of experience and AG with 12 years of experience). Recommendations for alternatives to any drug included in the IAHPC EML, but not present in an Indian EML, were taken from both experts individually in the first stage. Subsequently, both experts were invited to reach a consensus on any differences in recommendations in the second stage. The experts decided on alternative drugs after considering drug efficacy, safety, cost-effectiveness, availability in the Indian market and secondary effects of the drug that would be useful in a patient receiving palliative care. In situations where the drug recommended by IAHPC was considered efficacious, cost-effective and available in the Indian market, the experts did not offer any alternatives.

### Data analysis

To assess the adequacy of the lists, drugs present in the national and state EMLs were categorised as follows: all formulations present, some formulations present, no recommended formulation present and drug absent. We calculated the percentage of drugs present in each category in national and state EMLs using Equation 1. The different formulations that were mentioned for drugs included tablets, capsules, oral solutions, injectables, suppositories and salts.


$$\%\;\mathrm{of}\;\mathrm{drugs}\;\mathrm{available}\;\mathrm{in}\;t\mathrm{he}\;\mathrm{EML}\;\mathrm{for}\;\mathrm{the}\;\mathrm{category}\;=\;\frac{\mathrm{No}.\;\mathrm{of}\;\mathrm{drugs}\;\mathrm{present}\;\mathrm{in}\;t\mathrm{he}\;\mathrm{EML}}{\mathrm{Tota}l\;n\mathrm{umber}\;\mathrm{of}\;\mathrm{drug}s\;\mathrm{in}\;\mathrm{IAHPC}\;\mathrm{list}}\;x\;100\;(1)$$

We also looked at the adequacy of the EMLs to manage palliative care symptoms using alternative drugs identified using expert consensus and literature review as mentioned previously. The IAHPC EML mentions 16 common palliative care symptoms, which include depression, neuropathic pain, constipation, anorexia, nausea, vomiting, anxiety, mild to moderate pain, moderate to severe pain, delirium, terminal restlessness, terminal respiratory congestion, visceral pain, dyspnea, diarrhoea and insomnia. We assessed whether the national or state EMLs had at least one drug that could be prescribed for each of these indications. For diarrhoea, the presence of oral rehydration solution (ORS) in the list was considered necessary to adequately manage the symptoms. For terminal restlessness, we considered haloperidol essential for management, with midazolam as an add-on drug [30]. We reported this result as the proportion of EMLs that were adequate to manage all the aforementioned symptoms.

The most recent National Programme for Prevention and Control of Non-Communicable Diseases (NPNCD) guidelines evaluate access to palliative care by assessing morphine-equivalent consumption of strong opioid analgesics (except methadone) per cancer death [31]. Therefore, we specifically looked at the inclusion of various formulations of morphine (oral solution, oral tablet and injectable) in the EMLs as well. Subsequently, we calculated the proportion of EMLs, which included both oral and injectable morphine, only oral morphine, only injectable morphine and no formulation of morphine.

## Results

A total of 3 national and 27 state/UT EMLs were analysed in the study. Although India is a union of 28 states and 8 UTs, the remaining EMLs were unavailable in the public domain.

### Adequacy of drugs

**Figure 1.**
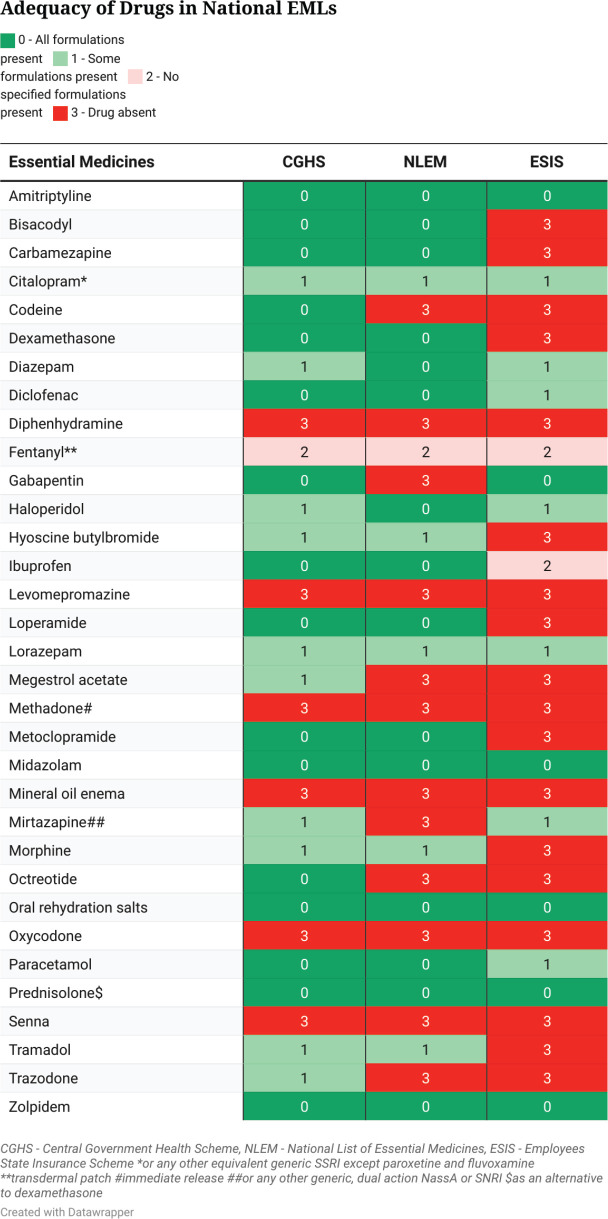
Adequacy of Indian national EMLs compared with the IAHPC EML for palliative care.

Among national EMLs, the CGHS had the highest number of all formulations of drugs (16 [48%]). Ten (30%) drugs had some formulations present, one drug had no recommended formulation present and six (19%) drugs were absent. The NLEM contained all formulations of 15 (46%) drugs, some formulations of 5 (15%) drugs, no recommended formulations of 1 (3%) drug and 12 (36%) drugs were absent. The ESIS EML had the least number of drugs with all formulations present (6 [18%]), some formulations were present of 7 (21%) drugs, no recommended formulations were present of 2 (6%) drugs and 18 (55%) drugs were absent (Figure 1).

**Figure 2.**
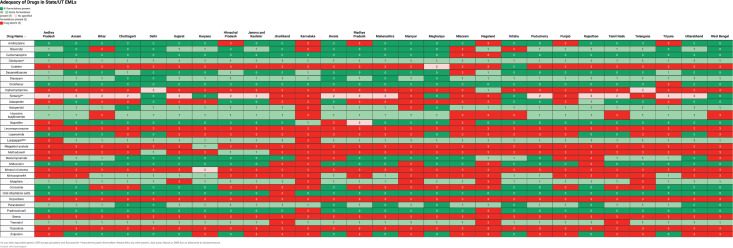
Adequacy of state/UT EMLs compared with the IAHPC EML for palliative care.

Among states and UTs, Delhi’s EML was the closest to the IAHPC EML and included all formulations required for 17 (52%) drugs. Eight (24%) drugs had some formulations present, one drug had no recommended formulation present, and seven (21%) drugs were absent. Karnataka’s EML included all recommended formulations of only 3 (9%) drugs, and some formulations of 6 (18%) drugs, while 24 (73%) drugs were absent. The adequacy analysis of all EMLs is presented in Figure 2 and Table 3. The presence of viable alternatives to absent drugs was variable across the EMLs, ranging from 4% of absent drugs in the Karnataka EML to 100% of absent drugs in CGHS, Delhi and Uttarakhand EMLs.

No Indian EML had all recommended formulations of morphine as oral solutions, tablets and injectables. Nationally, the CGHS EML and NLEM included morphine injectables and tablets, while the ESIS EML did not include morphine in any form. Nine (33%) state/UT EMLs included injectables and tablets. Twelve (44%) included only injectable morphine, and one (4%) included only tablets. Morphine was absent in five (16%) EMLs - Gujarat, Karnataka, Manipur, Nagaland and Telangana.

### Adequacy for symptom management

**Figure 3.**
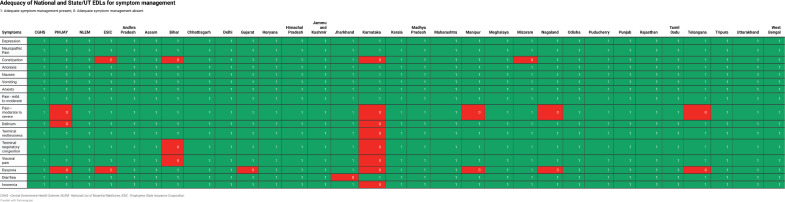
Adequacy of Indian EMLs for managing palliative care symptoms listed by IAHPC.

All of the 16 symptoms listed by IAHPC could be managed by at least one drug present in two (67%) national lists – CGHS and NLEM and 19 (70%) state/UT EMLs. The analysis for symptom management in EMLs is presented in Figure 3. Among the inadequately managed symptoms, constipation was not addressed by the EMLs of ESIC, Bihar, Karnataka and Mizoram. Moderate to severe pain management was insufficient in the EMLs of Karnataka, Manipur, Nagaland and Telangana. Manipur’s EML did not manage terminal restlessness effectively, Bihar’s EML did not manage both terminal respiratory congestion and visceral pain, while Karnataka’s EML did not manage any of these symptoms. Dyspnea was inadequately managed in the EMLs of ESIC, Gujarat, Karnataka, Manipur, Nagaland and Telangana. Jharkhand’s EML was the only one that left diarrhoea unmanaged, while the EML of Karnataka was the only EML inadequate to manage delirium and insomnia. However, depression, neuropathic pain, anorexia, nausea, vomiting, anxiety and mild to moderate pain were adequately managed by all state and national EMLs.

## Discussion

In our study, we report that India's national and state EMLs did not completely align with the recommendations of IAHPC. The Delhi EML was the closest to IAHPC recommendations, followed by the NLEM. Some drugs recommended by the IAHPC, such as levomepromazine, senna, trazodone and oxycodone, were absent from all state and national lists, except trazodone, which was present in the NLEM. Others, such as megestrol and mineral oil enema, were present in only one state list each, and only the NLEM included codeine and megestrol. While EMLs include fentanyl in the injectable form, the commonly prescribed formulation for pain management – transdermal patch, was included by only two states (Haryana and Meghalaya). The experts in the study felt that morphine was a more cost-effective and efficient substitute for transdermal fentanyl in the Indian setting due to the difficulty of titration and the high cost of the latter, which restricts access to it. Additionally, even though all EMLs, barring one, contained ORS for the management of diarrhoea, Indian EMLs were found to be inadequate to treat intractable diarrhoea since the presence of loperamide is integral for non-infective intractable diarrhoea management [32, 33]. Dyspnea, constipation and moderate to severe pain are other symptoms poorly managed by Indian EMLs. States such as Bihar, Karnataka, Telangana and the north-eastern states of Manipur, Nagaland and Mizoram lag behind the rest of the country in the inclusion of recommended drugs, as well as symptom management, even after including viable alternatives present in the EMLs.

Essential drugs cater to any population’s priority needs, availability and affordability. Given the rising number of patients needing palliative care, essential drugs must be made available and accessible to them. EMLs are instrumental in improving access to essential drugs through the public health system and reducing the financial burden on patients and caregivers for conditions that are relatively common in the community [34, 35]. The implementation of the National Essential Medicines Policy in China led to better prescription practices and a decline in average prescription costs. Another study looking at the impact of the essential drugs programme in peripheral health units in Yemen showed similar results, with improved availability and more rational use of drugs [36, 37]. Thus, limited global evidence highlights the impact of implementing EMLs in terms of improving access to treatment, enhancing drug availability, optimising prescription practices and reducing costs.

While we used the IAHPC EML, published in 2007, as the benchmark against which all other EMLs were compared, we noticed that certain updates based on recent evidence need to be incorporated. For example, in 2010, Fosbøl *et al* [38] reported an increased risk of cardiovascular events in patients receiving treatment with diclofenac which has been mentioned in the IAHPC list for the management of mild to moderate pain. Additionally, we propose a further classification of nausea and vomiting into that caused by gastroparesis, radiotherapy and chemotherapy to simplify prescription, as their treatments widely differ [39].

The increasing advocacy for fentanyl transdermal patches, touted for their potency in pain management, is raising concerns, particularly in low- and middle-income countries (LMICs) like India. While a 100-mg dose of injectable fentanyl is equivalent to 10 mg of morphine [40] in India, fentanyl costs 12 times as much as morphine per day. The high cost of fentanyl, coupled with its limited accessibility, makes it impractical for widespread use in LMICs [40, 41]. Indian EMLs did not include all the recommended formulations of morphine, a practical alternative to fentanyl. Morphine’s importance is highlighted by the fact that an objective of the National Program for Palliative Care (NPPC) is to increase its availability. However, while the IAHPC recommends the inclusion of oral morphine solution in addition to tablets and injectables, it is often not commercially available in India due to its short shelf life and limited manufacturers [42]. Hence, it may not be a feasible inclusion in an Indian EML for palliative care. Morphine usage is also used as an indicator to assess the coverage of palliative care services in the NPNCD [43]. Emphasis should be placed on ensuring that all formulations of morphine are available and affordable. Despite the 2014 amendment to the NDPS Act, which reduced the number of required licenses for opioid possession from six to one, complex regulatory procedures and a lack of prescription awareness among physicians continue to hinder patients' access to essential pain management medications like morphine. In 2014, India's total morphine consumption was a mere 278 kg. Considering that a patient with terminal cancer requires 75 mg of morphine per day for approximately 90 days, this amount is only sufficient to adequately treat 40,000 patients [44, 45]. Addressing these barriers is crucial for improving pain management in countries like India, where the need for affordable and effective pain relief is paramount. Simplifying regulatory processes and enhancing physician education on opioid prescriptions are key steps toward ensuring that patients receive the pain management they need.

The Sustainable Development Goal 3.8 mentions access to quality essential healthcare services and access to safe, effective, quality and essential medicines for the achievement of Universal Health Coverage (UHC). We noted the presence of different national EMLs, meant effectively for different sections of the population, with the CGHS EML meant for government employees performing better than the ESIS EML meant for other workers. This highlights redundancy in the system and a potential source for inequitable healthcare delivery. Additionally, the EMLs devised by individual states should ideally include all the drugs present in the national list, with the addition of drugs considered necessary based on the local epidemiology of diseases. Establishing a single national EML, aligned with global standards is integral to achieving UHC, especially for palliative care provision. By implementing EMLs tailored to include drugs required to deliver palliative care, healthcare systems can effectively address the diverse needs of patients while promoting equitable and cost-effective healthcare delivery [35].

India launched NPPC in 2012 to make high-quality palliative care accessible across all levels of health care [43]. This included making drugs for pain relief and other symptoms available at the primary healthcare level [46]. Thus, it is imperative that the benefits of drug inclusion in the EML are not restricted to tertiary care setups and that drugs are made available in primary and secondary care centres as well [43]. While national and state EMLs do not conform to the IAHPC recommendations, they contain alternatives for the management of symptoms commonly encountered in palliative care. However, some states, such as Bihar, Gujarat, Jharkhand, Karnataka, Mizoram, Manipur, Nagaland and Telangana, lack these alternatives, rendering them inadequate in managing some of these symptoms. It is crucial to incorporate essential medications from the IAHPC list or adopt alternatives to address these gaps. For instance, adding morphine to Nagaland’s EML would improve the management of both moderate to severe pain and dyspnea. Furthermore, Jharkhand should include ORS in its EML for the management of diarrhoea, as it is the only EML currently lacking this essential treatment.

Our findings highlight that there is a need for an EML tailored to palliative care in India and similarly in other countries, with drugs thoughtfully included as per existing procurement, storage and distribution constraints. EMLs specific for settings with different levels of available resources will help guide countries that currently do not have an EML or are looking to update existing EMLs.

## Strengths and limitations

Our study is the first to analyse the presence of specific medications essential for palliative care across the diverse EMLs of India. This contributes significantly to evaluating their readiness to manage the prevalent symptoms encountered by patients receiving palliative care. We were also able to delve deeper into analysing the management of common symptoms experienced by patients receiving palliative care in cases where the drugs recommended by the IAHPC were unavailable, and alternative options were explored.

Our study has a few limitations. First, while we identified the inclusion of medications for palliative care across multiple EMLs, we lacked the resources to assess their availability at the grassroots level and within hospitals. A comprehensive evaluation, combining our study with grassroots-level investigations, would provide a more accurate measure of access to essential drugs. Second, we did not compare the available drug doses in EMLs with the recommendations of IAHPC as the required dose can be attained by modifying the quantity consumed of the available drug. Finally, the costs of the alternate drugs listed by experts were not compared. However, it was a criterion that the experts considered when deciding the alternatives.

## Conclusion

Indian EMLs are not entirely in line with the IAHPC recommendations for essential palliative care drugs. However, they contain a range of drugs adequate to treat most symptoms requiring palliation. Considering the importance of morphine, both in palliative care symptom management and monitoring of palliative care-related national programs in India, the national and state/UT EMLs should be updated to incorporate oral and injectable formulations of morphine. There is a need to update the IAHPC list using recent evidence, and there is also a need to design a list based on different levels of available resources to guide countries in formulating their EMLs for palliative care service delivery.

## Conflicts of interest

The authors have no competing interests to declare.

## Funding

None.

## Ethical approval

The study did not involve any human or animal subjects and, therefore, did not require any ethical clearance.

## Data sharing

The data are available upon reasonable request that can be made to Dr Parth Sharma (Email ID: parth.sharma25@gmail.com).

## Author contributions

Corresponding author: PS

Joint authorship: DA and DS contributed equally to this paper.

Conceptualisation: PS; Reviewed study proposal: PS, SZ, MRR, AG; Data extraction: DA, DS; Data analysis: DA, DS; Scientific advisors: MRR, AG, SZ; Writing of the original draft: DA, DS; Review and editing of the final draft: All authors; Project supervision: PS, SZ.

## Figures and Tables

**Table 1.** National and state EMLs.

S. No.	National list	Year	Authorizing body
1	CGHS	Unknown	Directorate General of Health Services
2	ESIS	2010	Employees State Insurance Corporation (ESIC)
3	NLEM	2022	Ministry of Health and Family Welfare, Government of India
S. No.	State/UT	Year	Authorizing body
1	Andhra Pradesh	2013	Health and Family Welfare Department, Government of Andhra Pradesh
2	Assam	2023	Medical Education and Research Department, Government of Assam
3	Bihar	2022	Health Department, Government of Bihar
4	Chhattisgarh	2016	Department of Health and Family Welfare Department, Government of Chhattisgarh
5	Delhi	2022	Directorate of Health Services, Government of Delhi
6	Gujarat	2022	Government of Gujarat
7	Haryana	2013	Government of Haryana
8	Himachal Pradesh	2020	Health and Family Welfare Department, Government of Himachal Pradesh
9	Jammu and Kashmir	2022	Health and Medical Education Department, Government of Jammu and Kashmir
10	Jharkhand	Unknown	Government of Jharkhand
11	Karnataka	Unknown	Government of Karnataka
12	Kerala	2020	Government of Kerala
13	Madhya Pradesh	2020	Directorate of Health Services, Government of Madhya Pradesh
14	Maharashtra	2022	Commissionerate of Health Services, Government of Maharashtra
15	Manipur	2012	State Health Society, Manipur
16	Meghalaya	2023	Meghalayan Medical Drugs & Services Limited Health & Family Welfare Department Government of Meghalaya
17	Mizoram	2023	Mizoram Health and Family Welfare Department
18	Nagaland	2018	Health and Family Welfare Department, Government of Nagaland
19	Odisha	2020	Odisha Health and Family Welfare Department
20	Puducherry	2023	Department of Health and Family Welfare Services, Government of Puducherry
21	Punjab	2018	Government of Punjab
22	Rajasthan	Unknown	Government of Rajasthan
23	Tamil Nadu	2022	Government of Tamil Nadu
24	Telangana	Unknown	Department of Health, Medical and Family Welfare, Government of Telangana
25	Tripura	2017	Health and Family Welfare Department, Government of Tripura
26	Uttarakhand	2015	Department of Medical Health and Family Welfare, Government of Uttarakhand
27	West Bengal	2022	Department of Health and Family Welfare, Government of West Bengal

**Table 2.** Drugs identified for specific symptoms.

S. No.	Symptom	Drugs in IAHPC EML	Alternative drugs identified through literature review and expert consultation
1	Depression	Amitriptyline, Citalopram (or any other equivalent generic SSRI except paroxetine and fluvoxamine), Mirtazapine (or any other generic, dual action NassA or SNRI)	No alternative recommended
2	Neuropathic pain	Amitriptyline, Carbamazepine, Dexamethasone, Gabapentin	Pregabalin [17, 18]
3	Constipation	Bisacodyl, Senna	No alternative recommended
4	Diarrhoea	Codeine, Loperamide, Octreotide	Diphenoxylate [19]
		Oral rehydration salts	No alternative recommended
5	Pain – mild to moderate	Codeine, Diclofenac, Ibuprofen, Paracetamol, Tramadol	Naproxen [20]
6	Anorexia	Dexamethasone, Megestrol acetate, Prednisolone	No alternative recommended
7	Nausea	Dexamethasone, Metoclopramide, Diphenhydramine, Haloperidol, Hyoscine butylbromide	Ondansetron, Domperidone [21], Promethazine [22], Olanzapine
8	Vomiting	Dexamethasone, Metoclopramide, Diphenhydramine, Haloperidol, Hyoscine butylbromide, Octreotide	Ondansetron, [23] Domperidone, Promethazine, Olanzapine [24]
9	Anxiety	Diazepam, Lorazepam, Midazolam	No alternative recommended
10	Pain – moderate to severe	Morphine, Methadone, Fentanyl (transdermal patch), Oxycodone	No alternative recommended
11	Delirium	Haloperidol, Levomepromazine	No alternative recommended
12	Terminal restlessness	Haloperidol, Levomepromazine, Midazolam	No alternative recommended
13	Terminal respiratory congestion	Hyoscine butylbromide	Glycopyrrolate [25]
14	Visceral pain	Hyoscine butylbromide	Dicyclomine (only for colicky or spasmodic pain) [26]
15	Insomnia	Lorazepam, Trazodone, Zolpidem	Alprazolam, [27] Zaleplon, [28] Eszopiclone [29]
16	Dyspnea	Morphine	No alternative recommended

**Table 3.** Drug availability (%) compared with IAHPC recommendations.

S. No.	EML	All formulations present	Some formulations present	No recommended formulation present	Drug absent
National					
1	CGHS	48	30	3	19
2	NLEM	46	15	3	36
3	ESIS	18	21	6	55
States and UTs					
1	Andhra Pradesh	24	28	3	45
2	Assam	40	27	3	30
3	Bihar	30	27	3	40
4	Chhattisgarh	40	21	3	36
5	Delhi	52	24	3	21
6	Gujarat	33	27	3	37
7	Haryana	33	34	3	30
8	Himachal Pradesh	30	30	3	37
9	Jammu and Kashmir	30	30	3	37
10	Jharkhand	24	21	0	55
11	Karnataka	9	18	0	73
12	Kerala	39	22	3	36
13	Madhya Pradesh	24	24	7	45
14	Maharashtra	39	22	3	36
15	Manipur	27	18	0	55
16	Meghalaya	49	15	3	33
17	Mizoram	18	21	0	61
18	Nagaland	9	27	0	64
19	Odisha	42	25	0	33
20	Puducherry	33	31	3	33
21	Punjab	21	27	0	52
22	Rajasthan	33	28	3	36
23	Tamil Nadu	30	24	3	43
24	Telangana	27	27	3	43
25	Tripura	25	33	3	39
26	Uttarakhand	39	24	3	34
27	West Bengal	39	19	0	42

## Supplementary material

**Supplementary Table 1.** National and state EMLs.

S. No.	National list	Year	Authorizing body	Reference
1	CGHS	Unknown	Directorate General of Health Services	https://cghs.nic.in/REVISED%20LIST%20OF%20LIFESAVING%20DRUGS%20OF%20CGHS%20MSD%20DELHI.pdf
2	ESIS	2010	ESIC	https://www.esic.gov.in/Tender/rc135data.pdf
3	NLEM	2022	Ministry of Health and Family Welfare, Government of India	https://main.mohfw.gov.in/sites/default/files/Notification%20and%20Report%20on%20National%20List%20of%20Essential%20Medicines%2C%202022.pdf
**S. No.**	**State/UT**	**Year**	**Authorizing body**	**Reference**
1	Andhra Pradesh	2013	Health and Family Welfare Department, Government of Andhra Pradesh	https://cfw.ap.nic.in/pdf/Employees%20Data/Free%20D&D/2013HMF_MS204.pdf
2	Assam	2023	Medical Education and Research Department, Government of Assam	https://amscl.assam.gov.in/sites/default/files/public_utility/Notification%20No.MER_.424912-72%20-%20Essential%20Drug%20List%20%28EDL%29%20and%20Specialty%20Drug%20List%20%28SDL%29%202023-24_compressed.pdf
3	Bihar	2022	Health Department, Government of Bihar	https://shs.bihar.gov.in/SHS/Drug_Equipments/Sankalp_1729_12_Dt-08-12-2022-EDL-2022.pdf
4	Chhattisgarh	2016	Department of Health and Family Welfare Department, Government of Chhattisgarh	https://cgmsc.gov.in/Upload/EDL%202016.pdf
5	Delhi	2022	Directorate of Health Services, Government of Delhi	https://dgehs.delhi.gov.in/dghs/edl
6	Gujarat	2022	Government of Gujarat	https://gmscl.gujarat.gov.in/essential-drug-list.htm
7	Haryana	2013	Government of Haryana	https://hmscl.org.in/Edl.html
8	Himachal Pradesh	2020	Health and Family Welfare Department, Government of Himachal Pradesh	https://himachal.nic.in/WriteReadData/l892s/19_l892s/1593418077.pdf
9	Jammu and Kashmir	2022	Health and Medical Education Department, Government of Jammu and Kashmir	https://www.jkmsclbusiness.com/pdf/jkmscledl22jan22.pdf
10	Jharkhand		Government of Jharkhand	https://jmhidpcl.jharkhand.gov.in/uploads/Essential-Drug-List-Jharkhand.pdf
11	Karnataka		Government of Karnataka	https://mahitikanaja.karnataka.gov.in/Ksmscl/EssentialList?ServiceId=4380&Type=TABLE&DepartmentId=2118
12	Kerala	2020	Government of Kerala	http://kmscl.kerala.gov.in/KMSCL/uploads/announcements/DRUGLIST_2020-21_-_WEBSITE.pdf
13	Madhya Pradesh	2020	Directorate of Health Services, Government of Madhya Pradesh	https://www.sda.mp.gov.in/mphealth/en/drug-procurement/essential-drug-listpolicy
14	Maharashtra	2022	Commissionerate of Health Services, Government of Maharashtra	https://nrhm.maharashtra.gov.in/EDL.pdf
15	Manipur	2012	State Health Society, Manipur	https://nrhmmanipur.org/wp-content/uploads/2014/04/ANNEXURE-medicine-list-for-CHC-PHC-PHSC-UHC.pdf
16	Meghalaya	2023	Meghalayan Medical Drugs & Services Limited Health & Family Welfare Department Government of Meghalaya	https://meghealth.gov.in/docs/Essential_Drug_List_2023.pdf
15	Mizoram	2023	Mizoram Health and Family Welfare Department	https://health.mizoram.gov.in/uploads/attachments/2023/07/2d717add94f748f20debac8321ab6f06/essential-drugs.pdf
16	Nagaland	2018	Health and Family Welfare Department, Government of Nagaland	https://www.nhmnagaland.in/Notification_file_path/FACILITY%20TYPE%20ESSENTIAL%20DRUG%20LIST.pdf
17	Odisha	2020	Odisha Health and Family Welfare Department	https://osmcl.nic.in/sites/default/files/Essential%20Drug%20List%202020.pdf
18	Puducherry	2023	Department of Health and Family Welfare Services, Government of Puducherry	https://health.py.gov.in/revised-essential-drug-list-edl
19	Punjab	2018	Government of Punjab	https://phsc.punjab.gov.in/sites/default/files/2019-08/520183121639ListofEDL%26JSSKitems2018.xls
20	Rajasthan	Unknown	Government of Rajasthan	http://rmsc.health.rajasthan.gov.in/content/dam/doitassets/Medical-and-Health-Portal/rajasthan-medical-corporation/pdf/EDL/EDL%20final%20list%203.3.2020.pdf
21	Tamil Nadu	2022	Government of Tamil Nadu	https://tnmsc.tn.gov.in/user_pages/drugtender.php?drugcat=T21083
22	Telangana	Unknown	Department of Health, Medical and Family Welfare, Government of Telangana	http://tsmsidc.telangana.gov.in/open_record_view.php?ID=178
23	Tripura	2017	Health and Family Welfare Department, Government of Tripura	https://drive.google.com/file/d/10vH9hytgU6OM2o5gYhiiupJXGaL4x3Rs/view?usp=sharing
24	Uttarakhand	2015	Department of Medical Health and Family Welfare, Government of Uttarakhand	https://nhm.uk.gov.in/upload/tenders/Tender-11.pdf
25	West Bengal	2022	Department of Health and Family Welfare, Government of West Bengal	https://www.wbhealth.gov.in/uploaded_files/go/EDL_507.pdf

Not Available:

4 states: Arunachal Pradesh, Goa, Sikkim, Uttar Pradesh

5 UTs: Andaman and Nicobar Islands, Chandigarh, Dadra and Nagar Haveli and Daman and Diu, Ladakh and Lakshadweep
